# A Giant Cervical Decidual Polyp in Pregnancy Managed With Lactoferrin, Probiotics, and Ligation: A Case Report

**DOI:** 10.1002/ccr3.71996

**Published:** 2026-02-03

**Authors:** Satoshi Yoneda, Noriko Yoneda, Masami Ito, Kanto Shozu, Tatsuhiro Tsuda, Kazushige Sugie, Akitoshi Nakashima

**Affiliations:** ^1^ Department of Obstetrics and Gynecology University of Toyama Toyama Japan; ^2^ Clinical Laboratory and Transfusion Medicine Toyama University Hospital, A Cell Therapy Center Toyama Japan

**Keywords:** giant cervical decidual polyp, lactoferrin, ligation, pregnancy, probiotics

## Abstract

Giant cervical decidual polyps during pregnancy increase the risk of miscarriage and preterm delivery; however, optimal management has not yet been established. We report the case of a pregnant woman who presented at 11 weeks of gestation with a bleeding 4‐cm cervical polyp. Oral lactoferrin and probiotics ( *Clostridium butyricum* , *Enterococcus faecium* , and *Bacillus subtilis* ) were administered. The polyp stalk near the external cervical os was ligated with triclosan‐coated polydioxanone, resulting in necrosis and removal at 13 weeks, with complete resolution by 14 weeks. Vaginal *Lactobacillus* spp. increased and cervicitis resolved by 20 weeks without the use of antibiotics, despite the presence of *Ureaplasma* and/or *Mycoplasma* in vaginal secretions. The patient delivered a healthy infant at 40 weeks. This case highlights a minimally invasive strategy—polyp ligation combined with lactoferrin and probiotics—to help restore vaginal homeostasis, avoid the use of antibiotics, and achieve a favorable pregnancy outcome.

## Key Clinical Message

A minimally invasive approach combining cervical polyp ligation with oral lactoferrin and lactic acid–producing probiotics may help restore vaginal homeostasis and limit ascending infection in early pregnancy. This strategy may reduce the risk of spontaneous miscarriage or preterm delivery in patients with large decidual polyps while avoiding antibiotic use.

**Abbreviations**
sLMC/PTD: spontaneous late miscarriage or preterm delivery
sPTD: spontaneous preterm delivery

## 1 Introduction

Cervical polyps during pregnancy are associated with an increased risk of spontaneous late miscarriage or preterm delivery (sLMC/PTD), regardless of whether polypectomy is performed [1, 2, 3, 4, 5]. Decidual polyps, particularly those ≥ 12 mm, or with bleeding, are considered high‐risk for sLMC/PTD [1, 2], and extremely preterm delivery before 28 weeks remains a major concern [3, 4]. However, effective management strategies have yet to be established.

Although the mechanisms underlying sLMC/PTD in pregnancies complicated by cervical polyps remain unclear, it is hypothesized that the polyp itself induces local inflammation and/or facilitates ascending infection associated with bacterial vaginosis, a major cause of sPTD [6]. Recent studies have also highlighted the potential role of *Ureaplasma* and/or *Mycoplasma* in adverse pregnancy outcomes [7]. As intra‐amniotic co‐infections with *Ureaplasma*/*Mycoplasma* and other bacteria are detected in approximately 60% of sPTD cases occurring between 22 and 26 weeks of gestation [8], preventive measures targeting these pathogens are warranted.

While antibiotics may eradicate bacterial vaginosis during pregnancy, they have not been shown to significantly reduce the risk of sPTD [9]. Moreover, antibiotic effects are transient and may contribute to antimicrobial resistance [10]. In contrast, promoting vaginal homeostasis through the proliferation of *Lactobacillus* species may represent a safer and more sustainable preventive strategy. Lactoferrin has been reported to suppress the growth of *Gardnerella* and promote *Lactobacillus* proliferation [11, 12, 13], potentially preventing sPTD [13, 14, 15]. In addition, combined administration of *Lactobacillus* and lactoferrin has been shown to improve bacterial vaginosis [16, 17]. Given that pregnancies complicated by cervical polyps carry an increased risk of sLMC/PTD due to the potential for ascending infections, we believe that promoting vaginal *Lactobacillus* is at least essential for preventing such infections.

In high‐risk cases, such as giant or bleeding polyps [2], mechanical management may also be necessary to minimize inflammation and reduce infection risk. However, immediate surgical removal carries a risk of massive hemorrhage due to the thick stalk and abundant vascularity. A less invasive approach—ligation of the polyp stalk near the external cervical os to induce necrosis and delayed resection—may mitigate these risks. The use of sutures with antibacterial properties is recommended, because the thread may remain in the cervical canal and potentially enter the intra‐uterus [18].

We herein introduce a novel management strategy combining oral lactoferrin, probiotics containing *Enterococcus faecium* (a lactic acid–producing species), and polyp ligation using triclosan‐coated polydioxanone, which possesses antibacterial properties, to treat a pregnant woman with a giant cervical polyp and moderate genital bleeding, ultimately resulting in term delivery.

## 2 Case History/Examination

**FIGURE 1:**
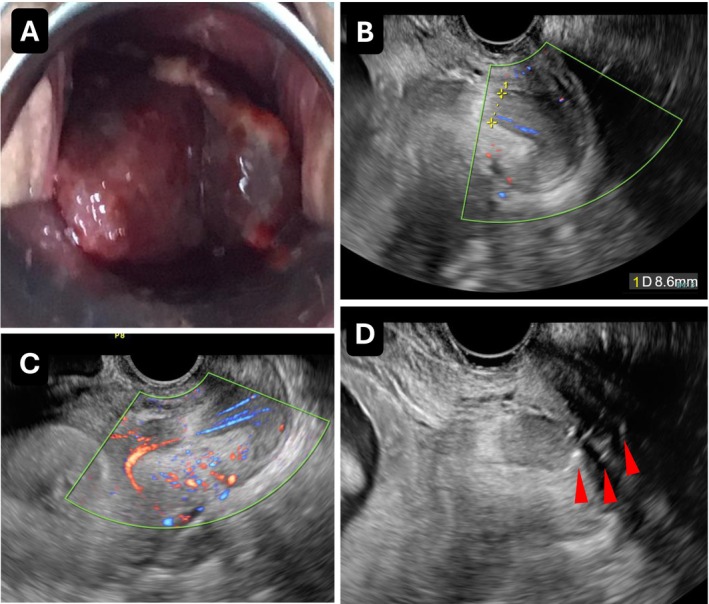
Initial presentation and ligation of a giant cervical polyp. (A) A giant cervical polyp measuring approximately 4 cm in diameter, occupying the vagina and obscuring the portio vaginalis uteri at 11 weeks and 5 days of gestation. (B) Transvaginal ultrasound revealing a thick polyp stalk measuring 8.6 mm in diameter. (C) Color Doppler ultrasound demonstrating blood flow within the polyp stalk. (D) Ligation of the polyp root near the external cervical os using triclosan‐coated polydioxanone sutures (three ligations; arrow).

A pregnant woman in her 30s, gravida 1, para 0, was referred to our hospital at 11 weeks and 5 days of gestation because of a giant cervical polyp with bleeding. All evaluations and treatments in this case were performed as part of routine clinical care with therapeutic intent and were not conducted as research. The polyp measured approximately 4 cm in diameter. It occupied the vagina and obscured the portio vaginalis uteri (Figure 1A). Transvaginal ultrasound revealed a thick stem (8.6 mm) with detectable blood flow in the endocervix, appearing to arise from the vicinity of the internal os, suggesting a giant decidual polyp (Figure 1B,C).

Vaginal culture identified *Lactobacillus* species (800 colonies), *Prevotella bivia* (20 colonies), *Alloscardovia* species (20 colonies), *Cutibacterium acnes* (5 colonies), and *Ureaplasma*/*Mycoplasma*. The cervical mucus interleukin‐8 (cIL‐8) concentration was markedly elevated at 1527.1 ng/mL (normal < 360 ng/mL [19]), indicating severe cervicitis.

## 3 Differential Diagnosis, Investigation and Treatment

The differential diagnosis included cervical malignancy, metastatic carcinoma to cervix, decidual polyp, endocervical polyp, Nabothian cyst, cervical leiomyoma, cervical fibroma, chronic cervicitis, tunnel cluster, and HPV‐related changes.

Considering the risk of massive hemorrhage, a staged approach was selected. Oral lactoferrin (300 mg/day) and probiotics containing *Clostridium butyricum* (10 mg/tablet), *Enterococcus faecium* (2 mg/tablet), and *Bacillus subtilis* (10 mg/tablet) were initiated, using a commercially available formulation approved for use in Japan.

On the following day, under informed consent, the polyp root near the external cervical os was ligated using triclosan‐coated polydioxanone sutures (three ligations) (Figure 1D). All procedures were performed in an outpatient setting.

**FIGURE 2:**
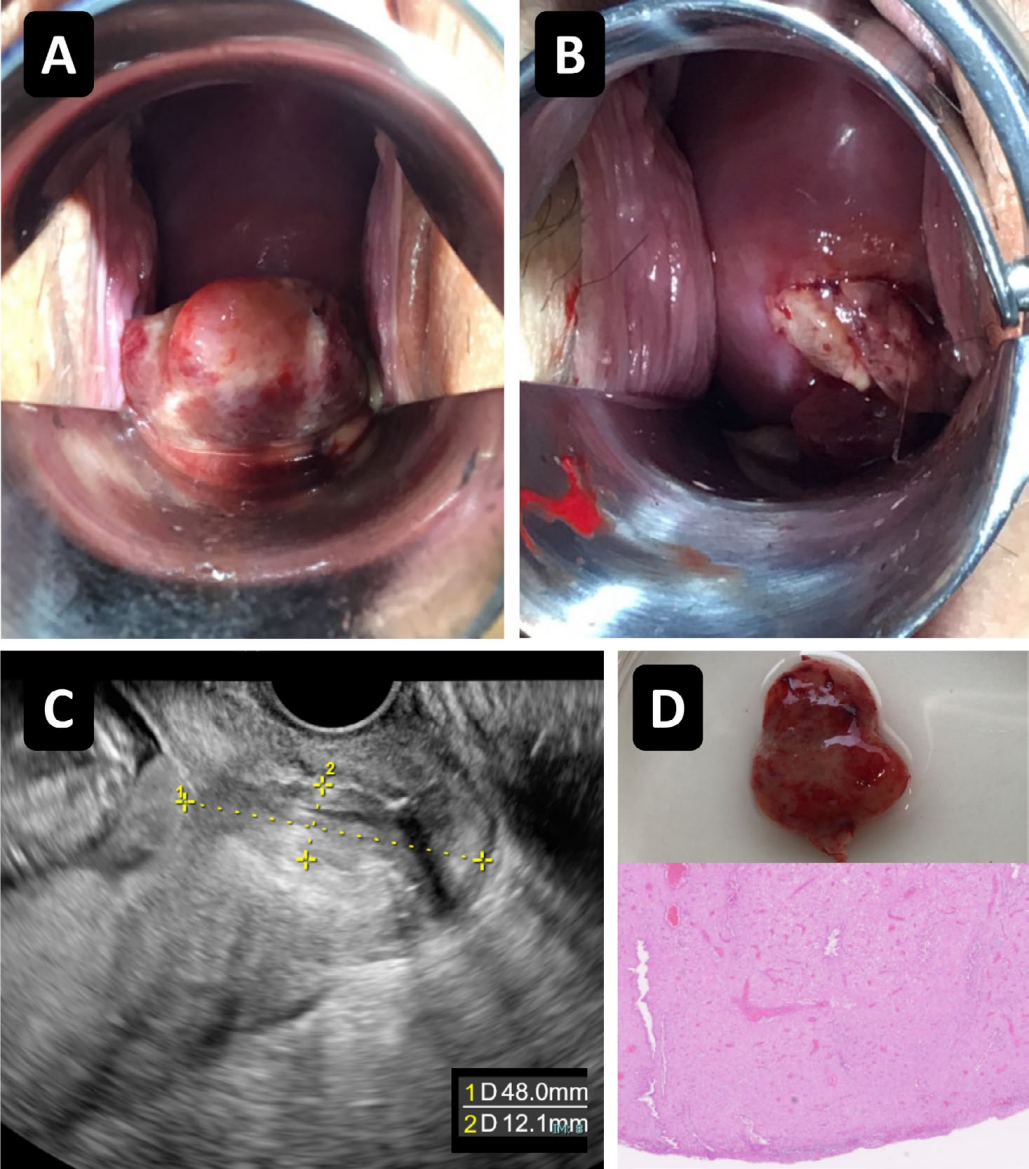
Polyp necrosis, spontaneous detachment, and histopathological findings. (A) Reduction in the size of the cervical polyp by 13 weeks and 6 days of gestation. (B) Gross appearance of the necrotic polyp after spontaneous detachment during an attempted repeat ligation, without bleeding. (C) Transvaginal ultrasound showing the maximum polyp length measuring 48 mm from the internal cervical os. (D) Spontaneously detached cervical polyp measuring approximately 15 mm in diameter, together with histopathological findings demonstrating decidualized stromal cells without evidence of malignancy, consistent with a decidual polyp (H&E stain, × 20).

By 13 weeks and 6 days of gestation, the polyp had decreased in size (Figure 2A,C). During an attempt at repeat ligation, the necrotic polyp detached and was removed without bleeding (Figure 2B,D). Histopathological examination confirmed a decidual polyp (Figure 2D).

**FIGURE 3:**
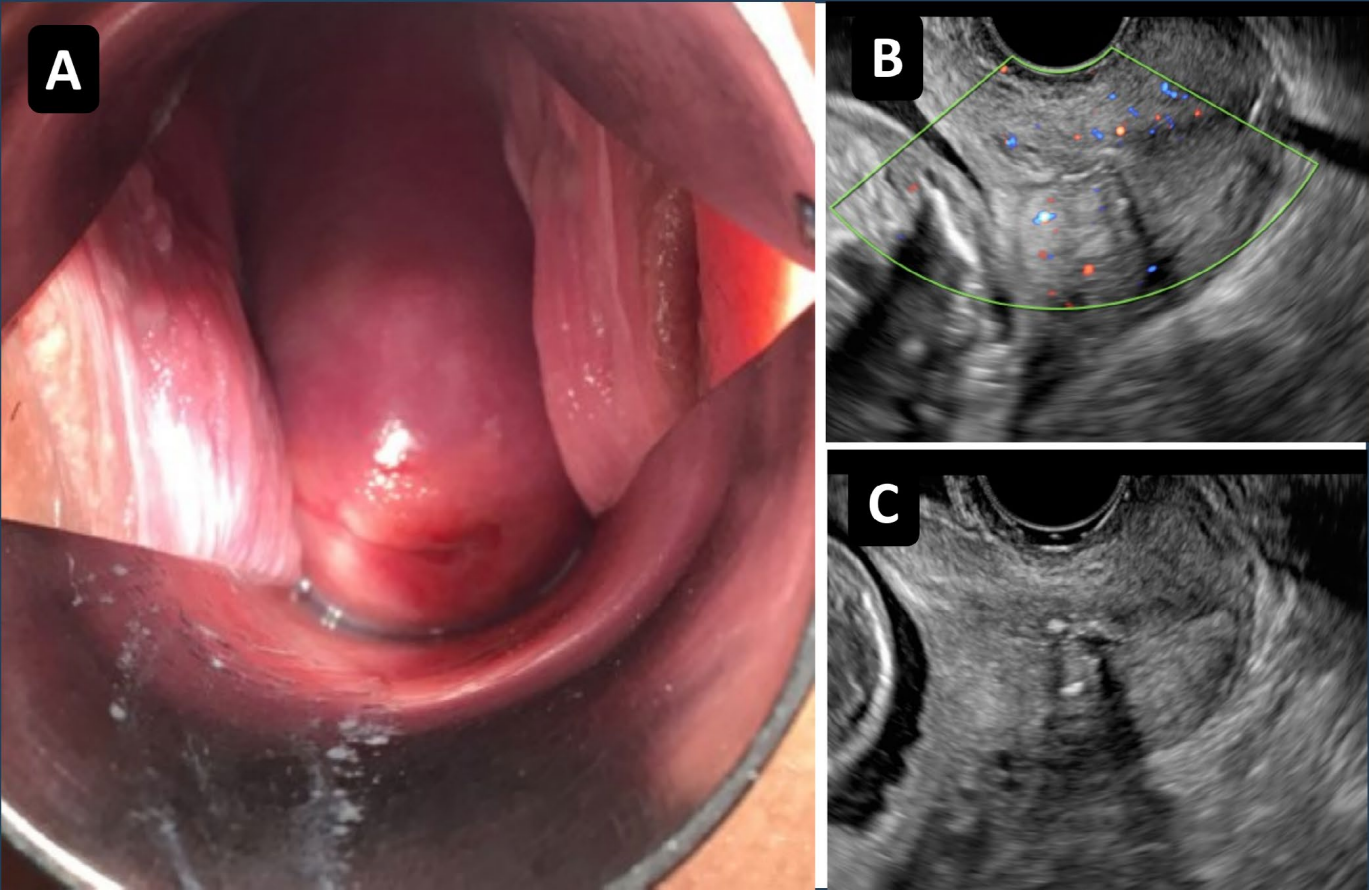
Follow‐up finding after complete resolution of the cervical polyp. (A) Complete disappearance of the cervical polyp at 14 weeks and 6 days of gestation. (B) Doppler ultrasound showing absence of blood flow in the cervical canal. (C) Triclosan‐coated suture remaining in situ within the cervix.

By 14 weeks and 6 days, the polyp had completely disappeared (Figure 3A), and ultrasound confirmed the absence of blood flow (Figure 3B). The triclosan‐coated suture remained in the cervix (Figure 3C). The patient reported no further clinical symptoms, such as genital bleeding.

## 4 Outcome and Follow‐Up

At 20 weeks and 6 days, the cIL‐8 concentration had decreased to 0.1 ng/mL, indicating resolution of cervicitis. Vaginal culture revealed *Lactobacillus* species (5000 colonies), *Finegoldia magna* (5 colonies), *Veillonella atypica* (3 colonies), and persistent *Mycoplasma*/*Ureaplasma*.

**FIGURE 4:**
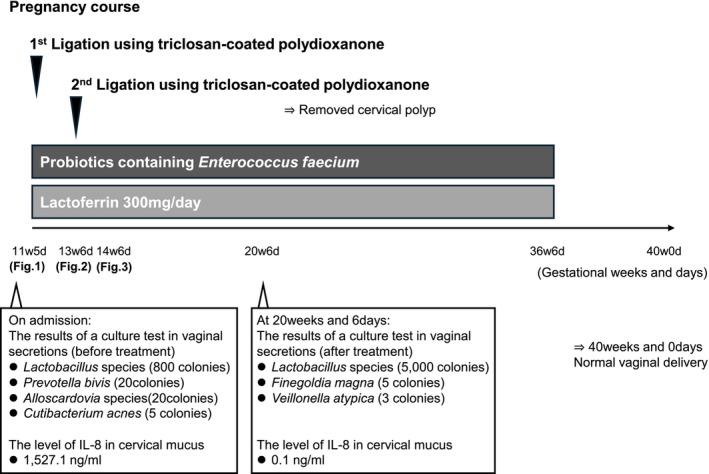
Pregnancy course. Combined treatment with lactoferrin, probiotics, and ligation may improve the vaginal and cervical environments. The proliferation of *Lactobacillus* species and resolution of cervicitis without antibiotics were important findings.

At 24 weeks and 6 days, the cervical length was 20 mm; however, she remained asymptomatic. Group B *streptococcus* screening at 36 weeks was negative. The patient delivered a healthy infant at 40 weeks and 0 days of gestation. Placental and umbilical cord pathology findings were unremarkable. Postpartum, both mother and baby remained well. A summary of the treatments, serial changes, and clinical courses during pregnancy is provided in (Figure 4).

## 5 Discussion

The positive predictive value of large cervical polyps (≥ 12 mm) with genital bleeding for sLMC/PTD before 34 weeks of gestation has been reported to be 63.6% [2]. In the present case, although the individual contributions of lactoferrin, probiotics, and ligation using triclosan‐coated polydioxanone sutures cannot be definitively determined, several clinical improvements were observed: *Lactobacillus* colonies increased from 800 to 5000, cIL‐8 decreased markedly from 1527.1 to 0.1 ng/mL, and severe cervicitis resolved by 20 weeks. These findings suggest the prevention of ascending infection and/or inflammation. While spontaneous improvement cannot be entirely excluded, the severe cervicitis in this case was likely attributable to the giant cervical polyp and paucity of vaginal *Lactobacillus*; therefore, the improvement was considered at least partly related to our therapeutic approach.

Vaginal cultures were performed using one liquid medium for the detection of *Ureaplasma* and/or *Mycoplasma*, and four agar media for the detection of other bacteria. Because culture‐based testing cannot detect all microorganisms and bacterial loads were interpreted semi‐quantitatively, the findings should be interpreted with caution. Nonetheless, the marked increase in vaginal *Lactobacillus* following administration of probiotics and lactoferrin was notable.

Lactoferrin has demonstrated efficacy in refractory bacterial vaginosis [13] and may help to prevent sPTD [13, 14, 15]. Furthermore, the combination of *Lactobacillus*‐containing probiotics and lactoferrin has been reported to reduce recurrent bacterial vaginosis [20]. Thus, restoring and maintaining vaginal homeostasis through *Lactobacillus* proliferation, rather than relying solely on antibiotics, may be important, particularly in pregnancies at increased risk for ascending infection. In this case, the combination of *Enterococcus faecium* –containing probiotics and lactoferrin may have contributed to the improvement. Given the projected rise in mortality due to antimicrobial‐resistant bacteria by 2050, strategies that minimize unnecessary antibiotic use remain clinically meaningful [10].

We previously reported a case in which a giant cervical polyp was successfully managed with probiotics alone [21]; however, we subsequently encountered a similar case that resulted in late miscarriage. These contrasting experiences highlight the need for additional or more comprehensive strategies in the management of high‐risk cervical polyps during pregnancy.

Surgical removal of large cervical polyps during pregnancy carries a risk of heavy bleeding. Because slippage of the ligature after immediate resection may cause massive hemorrhage, we avoid this commonly used method. A less invasive approach—ligation of the stalk near the external os to induce necrosis followed by delayed removal—may reduce these risks. In the present case, ligation effectively decreased blood flow, allowing safe removal of the necrotic polyp. Although this strategy requires additional time and may induce mild inflammation, it substantially reduces the risk of hemorrhage and can be performed on an outpatient basis. As expected, the ligated thread persisted within the cervical canal [18], underscoring the need for an antibacterial suture. A residual stalk may have remained after shedding; however, once its vascular supply is eliminated, spontaneous regression is likely.

Two approaches were employed in this case: (i) restoration of the vaginal microbiota with probiotics and lactoferrin, and (ii) polypectomy using a ligation‐necrosis technique. Although it is difficult to determine which intervention was primarily responsible for the favorable clinical course, improving vaginal flora appears particularly important. In high‐risk cases such as giant cervical polyps, a minimally invasive ligation‐necrosis approach may be beneficial when combined with measures that enhance vaginal *Lactobacillus*.

The patient reflected on her experience as follows: “Although I felt anxious, I followed my doctor's recommendations with a positive attitude, and my baby was ultimately born safely at term. I sincerely hope that my experience will help other mothers and infants facing the risk of preterm birth due to giant cervical polyps.”

Overall, this case suggests that combining a minimally invasive ligation‐necrosis approach with measures to restore vaginal homeostasis—such as probiotics and lactoferrin—may help reduce inflammation, avoid unnecessary antibiotic use, and support a favorable pregnancy outcome. Further accumulation of similar cases is needed to clarify the safety and effectiveness of this approach.

## Author Contributions

**Satoshi Yoneda:** conceptualization, data curation, resources, visualization, writing – original draft, writing – review and editing. **Noriko Yoneda:** data curation, writing – original draft, writing – review and editing. **Masami Ito:** writing – review and editing. **Kanto Shozu:** visualization, writing – review and editing. **Tatsuhiro Tsuda:** writing – review and editing. **Kazushige Sugie:** investigation, resources, writing – review and editing. **Akitoshi Nakashima:** writing – review and editing.

## Funding

The authors have nothing to report.

## Ethics Statement

The authors have nothing to report.

## Consent

Written informed consent was obtained from the patient for publication of this case report and the accompanying images.

## Conflicts of Interest

The authors declare no conflicts of interest.

## Data Availability

The authors have nothing to report.
